# The Emotional Mechanisms of Interpersonal Preemptive Behavior

**DOI:** 10.3389/fpsyg.2022.841960

**Published:** 2022-03-17

**Authors:** Lei Liu, Xiyan Song, Yu Li

**Affiliations:** Department of Psychology, College of Teacher Education, Ningbo University, Ningbo, China

**Keywords:** preemptive behavior, interpersonal interaction situation, emotional mechanisms, fear, hope

## Abstract

Interpersonal preemptive behavior means that a party undertakes a costly action that inflicts harm to another to remove or disable a potential threat. This present study examined the emotional mechanisms underlying interpersonal preemptive behavior. The findings revealed that in interpersonal interaction situations, individuals experienced higher levels of fear and hope when they perceived the potential threat of the gaming partner and were more likely to initiate preemptive behavior; fear and hope both mediated the relationship between potential threat and preemptive behavior, but they had opposite effects, with fear increasing individuals’ preemptive behavior, while hope decreases individuals’ preemptive behavior. This study has important theoretical implications for a deeper understanding of the causes of interpersonal conflict.

## Introduction

Preemptive behavior is defined here as instances in which a party undertakes a costly action that inflicts harm to another to remove or disable a potential threat (Halevy, 2017). The behavior occurs not only in high-stakes, competitive interaction situations, such as those involving political adversaries, business rivals, or armed forces (Fearon, 1995), but also in interaction situations in numerous life domains. For example, a child on the playground shoves another child whom he thought was out to get him; a girl breaks up with her boyfriend to make sure he does not break up with her; an employee quits the job to avoid being fired. In these situations, in order to protect themselves from the other person, people tend to adopt a “preemptive behavior” when dealing with interpersonal conflict, reducing or eliminating their own perceived potential threat. These suggest that preemptive behavior is common in daily life.

Ancient Chinese thinkers have long been aware of the significance of preemptive strategies in resolving interpersonal conflicts. For example, ancient Chinese books recorded that “Strike first or be struck by others later,” meaning that the first to strike is in a position to take the initiative, while the second to strike is controlled by others. Now the strategic belief of “first strike is the best” is also commonly held by humans when faced with potential threats. In this regard, preemptive behavior has a positive aspect in conflict resolution. However, preemptive behavior occurs before either party has attacked the other, and it is often uncertain or ambiguous whether the other party does pose a threat to them. That means independent of the opponent’s intention to attack, an attack can reinforce existing hostility or provoke new hostility, leading to a deterioration in the relationship between the interacting parties, which in turn leads to more aggressive behavior and with costly consequences for both parties. Base on this, preemptive behavior is not conducive to a good relationship. Therefore, it is of great relevance to explore preemptive behavior. This study attempts to examine the emotional mechanisms of preemptive behavior from interpersonal level.

So, when people perceive a potential threat of aggression from another person, do they tend to preemptive behavior before they are attacked? To answer this question, Simunovic et al. (2013) designed the Preemptive Strike Game (PSG), which is based on a modification of a two-player economic game. In this game, in order to prevent a greater loss of their vested interest, two players can attack each other by quickly pressing a red button on a computer screen. The researchers compared the results of the unilateral condition (only one player had the opportunity to press the red button) with the bilateral condition (both players had the opportunity to press the red button). The results showed that 50% of the participants chose to press the red button in the bilateral condition, while only 4% in the unilateral condition, which was significantly lower than the former. That means people are more likely to behave preemptively when they perceive a potential threat to their own vested interests. In addition, the researchers (Simunovic et al., 2013) presumed, using an additional PSG, that participants may have acted preemptively against the game opponent for defensive purposes out of fear.

Situations that trigger preemptive behavior involve uncertainty, risk and ambiguity (Fox and Weber, 2002), leading individuals to develop mixed affective states, which include both negative and positive affective states (Halevy, 2017). Halevy (2017) suggests that the emotional perspective of preemptive behavior based solely on fear is simple incomplete and should be extended to the effects of other specific emotions. He explored the effects of five emotions (i.e., fear, anger, disgust, happiness, and hope) on preemptive behavior, and found that hope significantly reduced preemptive behavior, whereas none of the other emotions had this effect. This result suggests that hope may have a unique role in reducing preemptive behavior. Overall, Simunovic et al. (2013) interpreted individuals’ initiation of preemptive behavior as defensive aggression based on fear, but only reasoned the relationship between threat and fear based on previous researches (Schelling, 1980; Andreoni, 1995) instead of measuring the fear. On the other hand, Halevy (2017) found that hope was effective in reducing preemptive behavior without obtaining direct evidence that fear motivated the preemptive behavior. So, does fear play a role in preemptive behavior? How likely is it that hope and fear co-exist? In this regard, there are no consistent conclusion on the emotional mechanisms of interpersonal preemptive behavior. Now there are only two studies which have examined interpersonal preemptive behavior from psychological perspective. Therefore, the aim of this study is to examine the role of emotions in preemptive behavior using an adaptation of the interpersonal PSG.

Previous research found that situations including threat and uncertainty automatically induce fearful emotion (Gullone, 2000; Jarymowicz and Bar–Tal, 2006). However, hope as a positive future-oriented emotion with a sense of uncertainty may also be present in individuals’ responses to these situations (Lazarus, 1999). Hope emerges when people are “fearing the worst but yearning for better and believing the wished-for improvement is possible” (Lazarus, 2006, p. 16), which means feeling of hope is often mixed with feeling of fear. Recently, researchers found that hope positively predicted conciliatory attitudes and support for peace in the context of the Israeli–Palestinian conflict (e.g., Cohen-Chen et al., 2014a,^2015^). The line of research demonstrated that hope can have a positive effect in an affectively complex, threatening and uncertain real-world situation, and that the effects of hope on psychological processes in this context are distinct from the effects of other negative emotions (fear: Cohen-Chen et al., 2014b).

We argue that in real-world interpersonal interactions, initiating preemptive behavior requires consideration of the benefits and drawbacks of people’s decision-making behavior for the preservation of their vested interests. Thus, when performing interpersonal PSG, these emotions (e.g., fear and hope) are likely to influence preemptive strikes in opposite directions. Whereas fear’s negative valence is likely to facilitate preemptive behavior, hope’s positive valence is likely to inhibit preemptive behavior. Accordingly, we predict that (1) in interpersonal interaction situations, participants are more likely to initiate preemptive behavior when they perceive a potential threat from the opponent than when they do not perceive that; and (2) emotional experiences (fear and hope) induced by potential threats play a mediating role in preemptive behavior. Specifically, fear has a positive indirect effect, while hope has a negative indirect effect.

## Materials and Methods

### Participants and Design

The study was one-way between-subjects experimental design. The software G*Power 3.1 was used to estimate the sample size before the experiment. To obtain a medium effect size (*w* = 0.3), with the α level set at 0.05 and the statistical power set at 0.8, the calculated sample size was about 88 individuals. A total of 151 university students participated in the PSG under two experimental conditions: bilateral condition and unilateral condition. Because the nature of the experiment required an even number of participants, a confederate was used in cases of cancelation. Eleven participants were found not to have read the instructions carefully or not to have understood the rules of the game and were therefore excluded (7.28%). After excluding invalid data, valid data remained for 140 (68 females) participants aged 18–25 years (*M* = 21.77, *SD* = 2.29), 71 in the unilateral condition and 69 in the bilateral condition. All participants had normal or corrected–to–normal vision, reported no history of neurological diseases or affective disorder, volunteered to participate in the experiment and signed an informed consent form. At the end of the experiment, we exchanged each participant’s remaining game coins (each worth RMB 0.1) for RMB as their payment for the experiment.

### Procedure

The participants were invited to the lab to complete the games, then experimenter randomly assigned them to bilateral condition and unilateral condition by drawing sticky notes (2 conditions: bilateral condition and unilateral condition) from the table. Everyone faced a computer surrounded by partitions that prevented others in the room from seeing him/her. They need to read the instructions and complete the PSG which was presented in Z-tree (Fischbacher, 2007), the role of task (Player A or B) was also randomly assigned by Z-tree. They had a communal countdown to ensure they have the same status when facing the preemptive behavior situation (Simunovic et al., 2013).

#### Bilateral Preemptive Strike Game

Two players (A/B) in different rooms were involved in the task alone (neither knows the true identity of the other). The instructions stated that each player has 150 game coins as capital and can decide independently how to use it, they are asked to protect their capital from loss by pressing or not pressing the “red button.” Pressing the “red button” means that the participant prevents the other player from causing loss to their capital, while not pressing means that the participant forfeits the opportunity to stop the opponent. Both players have 60 s to make a decision (a 5 s countdown is provided before) and the game would be played only once. The rules of the game are shown in Table 1.

**TABLE 1:** The rule of bilateral PSG.

Choice		B	
		**Waiting**	**Red button**
A	Waiting	A and B each loses 0 game coins	1. A loses 100 game coins 2. B loses 10 game coins
	Red button	1. A loses 10 game coins 2. B loses 100 game coins	1. Fast player (A/B) loses 10 game coins 2. Slow player (A/B) loses 10 game coins

### Unilateral Preemptive Strike Game

The basic structure and rules of the game are same as Bilateral PSG, except that only player A can choose between pressing or not pressing the “red button” in the game, and player B do not have “red button,” that means he can only wait for 60 s. The rules of the game are shown in Table 2.

**TABLE 2:** The rule of unilateral PSG.

Choice		B (no red button)
A	Waiting	A and B each loses 0 game coins
	Red button	1. A lose 10 game coins; B lose 100 game coins

After completing the PSG (but before the participants were informed of the results of the experiment), the participants were asked to complete the emotional questionnaire. Participants reported their feelings in response to the following question: “I am worried that the other person in the game will press the red button and make me suffer more” and “I hope that the other person in the game will not press the red button to prevent me from suffering more losses” (11-point scale: 0 = not at all, 10 = very strong).

Following the study of Simunovic et al. (2013), participants were asked to complete an additional PSG after completing a Bilateral (or Unilateral) PSG. All participants were given an additional “blue button,” and they could press the red button, the blue button, or neither. Pressing the red button had the same effect as in the former experiment; pressing the blue button reduced both players’ respective rewards by 10 coins and disabled further attacks. In all conditions, the participants had only one opportunity.

After completing the additional PSG, the participants were asked to complete the same emotional questionnaire again. Then participants received the results of the game and experimental remuneration.

## Results

### Preemptive Behavior

#### Preemptive Strike Game

**FIGURE 1:**
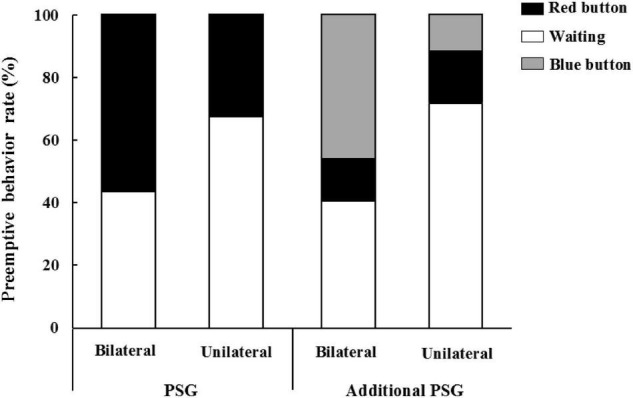
The effect of bilateral/unilateral condition on preemptive behavior.

Those individuals in bilateral condition were significantly more likely to press the red button against their counterparts (56.52%) than participants in the unilateral condition (32.39%), *x*^2^ = 8.26, *p* = 0.004 (please seeing Figure 1). This is consistent with Simunovic et al.’s (2013) results (Study 1), suggesting that individuals responded to threat of being attacked by launching preemptive behaviors.

#### Additional Preemptive Strike Game

The results showed that the main effect of different condition on the three behaviors was significant, *x*^2^ = 21.50, *p* < 0.001. A two-by-two comparison using the Bonferroni method showed that there was no significant difference between bilateral condition (13.04%) and unilateral condition (16.90%) with the proportion of pressing the red button. Participants who pressed the blue button in bilateral condition (46.38%) was significantly higher than those in unilateral condition (11.27%). Participants who chose waiting in bilateral condition (40.58%) was significantly lower than those in unilateral condition (71.83%; please seeing Figure 1). These results suggest that in interpersonal interaction situations, individuals prefer to press the blue button in order to protect their interests from (less) loss when they perceived potential threats from the other party.

### Emotional Mechanisms

**FIGURE 2:**
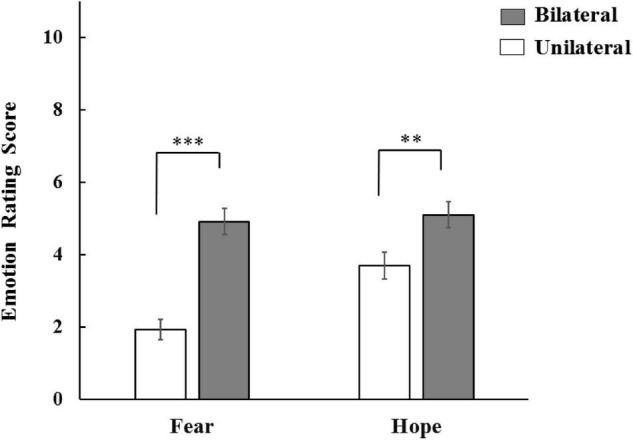
The effect of bilateral/unilateral condition on fear and hope; error bars depict standard errors; **p < 0.01 and ***p < 0.001.

Participants in bilateral condition reported feeling significantly higher level of fear (*M* = 4.91, *SD* = 2.95) than those in unilateral condition (*M* = 1.92, *SD* = 2.38), *t* (138) = 6.60, *p* < 0.001, *d* = 1.12, CI [2.100, 3.896]. Participants in bilateral condition reported feeling significantly higher level of hope (*M* = 5.12, *SD* = 3.03) than those in unilateral condition (*M* = 3.70, *SD* = 3.13), *t* (138) = 2.71, *p* = 0.008, *d* = 0.46, CI [0.383, 2.441], indicating that perceiving the potential threat of the other person was effective in increasing the individuals’ level of fear and hope (Figure 2).

Mplus 7.11 software was used to examine the correlation between emotion (fear and hope) and preemptive behavior, and logistic regression analysis was conducted on the experimental data using two different emotion rating scores (continuous variable) as predictor and preemptive behavior (categorical variable) as dependent variable. The results (Table 3) showed that fear positively predicted preemptive behavior, *p* < 0.001, and hope did not predict preemptive behavior, *p* = 0.106. The results indicated that when perceiving a potential threat from the opponent, the higher the participant’s fear, the more likely he or she was to initiate preemptive behavior, whereas hope did not have this effect.

**TABLE 3:** The logistic regression model of emotion on preemptive behavior.

Variable	Predictor	*B*	*SE*	*p*	*LLCL*	*ULCL*
Preemptive behavior	Fear	0.38	0.04	0.000	0.061	0.206
	Hope	−0.17	0.03	0.106	−0.125	0.011

To further elucidate the emotional mechanisms in interpersonal preemptive behavior, a bias-corrected Bootstrapping test (5,000 draws) was conducted using Mplus 7.11. In the mediational model of this study, both the independent variable (bilateral/unilateral condition) and the dependent variable (preemptive behavior) were categorical variables, and the mediating variables fear and hope were continuous variables. In order to align the scales of the linear and logistic regressions, a *z*-transformation was done on the regression coefficients before the mediating effects test (Iacobucci, 2012), and thus the mediating effects only report the mediating effect values and 95% Bootstrap confidence intervals (Fang et al., 2017).

From Table 4, when the unilateral condition was used as the reference, the mediating effect of bilateral condition on preemptive behavior through fear was 0.22, with a 95% Bootstrap confidence interval of [0.101, 0.339], excluding “0” indicating that the mediating effect was significant; Meanwhile, the mediating effect of bilateral condition on preemptive behavior through hope was −0.09, with a 95% Bootstrap confidence interval of [−0.167, −0.004], excluding “0” indicating that the mediating effect was significant; After adding fear and hope as the mediating variables, the direct effect of bilateral condition on preemptive behavior was 0.16, with a 95% Bootstrap confidence interval of [−0.047, 0.373], including “0” indicating that the direct effect was no longer significant. The results suggest that both fear and hope mediate the effecting of bilateral condition on preemptive behavior, but that the two have opposite effects. Specifically, fear increases preemptive behavior, whereas hope decreases preemptive behavior.

**TABLE 4:** Mediational model wherein fear and hope underlie the effect of bilateral/unilateral condition on the preemptive behavior.

Mediation path	Estimated value	95% CI	
		
		Low	High
Using unilateral condition as reference:			
Bilateral condition→ Fear→ Preemptive behavior	0.22^a^	0.101	0.339
Bilateral condition→ Hope→ Preemptive behavior	−0.09^a^	−0.167	−0.004
Bilateral condition→ Preemptive behavior	0.16	−0.047	0.373

*^a^indicates that the mediating effect is significant.*

## Discussion

This study examined the emotional mechanisms of interpersonal preemptive behavior using a PSG. The findings revealed that in interpersonal interaction situations, individuals chose to act preemptively when they were aware of the potential threat of the other party, as opposed to when they were not. Fear and hope mediated the causal relationship between threat and preemptive behavior, but their effects were reversed, with fear increasing the preemptive behavior and hope decreasing the preemptive behavior.

The present study confirmed previous findings (Simunovic et al., 2013; Halevy, 2017) that individuals were more likely to attack preemptively when they perceived a potential threat to their vested interests. This suggests that potential threats are a key factor in inducing preemptive behavior in preemptive situations, where individuals’ need for safety compels them to initiate attacks (Schelling, 1980; Simunovic et al., 2013). Furthermore, in the additional PSG, perception of a potential threat from the opponent in the game increased the proportion of people pressing the blue button relative to not perceiving a potential threat, which indicated that people would press the blue button in order to protect their interests; Moreover, it was found that people did not want to attack the other person in order to cause them greater damage, but rather to protect their own vested interests in this way by comparing the participants’ behavior in the PSG with those in the additional PSG.

Importantly, this study found that individuals’ perceptions of potential threat from the opponent of the game were effective in increasing participants’ levels of fear and hope. But that the two had opposite effects, specifically, fear increased preemptive behavior and hope decreased preemptive behavior. This suggests that “fear” and “hope” are not two opposite dimensions of the same subject, but two separate dimensions. Based on the “affect heuristic theory,” individuals relied on their subjective emotional experiences arising from the situation to make decisions (Slovic et al., 2007; Dohle et al., 2010). For example, when individuals perceived danger in their environment, the situation automatically triggered fear in them (Gullone, 2000; Jarymowicz and Bar–Tal, 2006), then further influenced their behavioral responses. Previous research found that fear motivated aggression in social conflict situations (Bar–Tal, 2001). Studies from animals also found that in predatory situations, prey relied on fear signals originating from the brain’s amygdala to act defensively against predators (De Dreu et al., 2015). In present study, the preemptive situation was characterized by threat, uncertainty and ambiguity (Fox and Weber, 2002). Although neither interacting individuals had the intention of initiating a surprise attack, they were fear, which led to suspicion and defensive aggression. In addition, according to previous research (She et al., 2016, 2017; Song et al., 2021), fear could reduce individuals’ patience in intertemporal choice, leading to a “short-sighted tragedy,” where people perceived larger benefits in the long term as more risky than smaller benefits in the short term, and therefore tended to forego greater future rewards. For this reason, the experience of fear in preemptive situation could lead to lack of patience and self-control, with individuals choosing to attack others to protect their own interests immediately, rather than giving up the attack and waiting the greater benefits.

As discussed above, situations that inducing preemptive behavior were characterized by threat, uncertainty and ambiguity (Fox and Weber, 2002), which not only automatically triggered fear (Gullone, 2000; Jarymowicz and Bar–Tal, 2006), but also triggered positive future-oriented emotions with uncertainty, such as hope (Lazarus, 1999). Hope was a positive emotion that arose from thinking about expected future (Frijda, 1986) and gave people confidence in achieving their future goals (Snyder, 2000). Researches have shown that experiencing hope in a conflict context was associated with people’s support for peaceful policies and actions (Halperin and Gross, 2011; Cohen-Chen et al., 2015). This suggests that experiencing hope enables people to resolve conflict in a peaceful manner. This is in line with the findings of this present study that when people are in a preemptive situation, hope reduces preemptive behavior.

The present study makes a contribution to social conflict, emotions and interactive decision-making. First, the findings deepen our understanding of social conflict by showing that aggressive behavior can occur in interpersonal interaction situations even in the absence of motivation to attack and when both parties benefit from maintaining the *status quo*. From game theory perspective, a preemptive situation is a low-conflict situation in which both parties can maximize their own interests at the same time; however, most participants prefer to protect their own interests by compromising a certain number of available resources in order to engage in preemptive behavior. Thus, the fact that preemptive behavior can occur in “benign” situations, which poses an important theoretical and practical problem that will require the attention of researchers. Secondly, this study has deepened our understanding of the role of emotions in decision-making. The results of this study found that interpersonal preemptive situations induce strong feelings of fear and hope, which in turn influence preemptive behavior, suggesting that future research needs to look not only at the influence of cognitive factors on interpersonal interaction decisions, but also at the role of emotional factors in them.

The present study also has limitations. First, this study partially adapted the interpersonal PSG designed by foreign scholars to suit Chinese participants, future studies may try to adopt other paradigms and larger sample size to verify the reliability. Second, this study examined participants’ levels of fear and hope using only one emotion self-rating item separately. Although self-report questionnaire is a great method to examine the role of emotions in decision-making (Ding et al., 2014; Todd et al., 2015; Ho et al., 2016; Song et al., 2021), future research should use several items per dimension, instead of the single-item measures of hope and fear, which would deepen the participants’ understanding of their own emotional experiences and allow for more realistic reporting. Thirdly, the psychological mechanisms of interpersonal preemptive behavior involve both emotional and cognitive aspects. This study only examined the role of emotions, and future research could examine the influence of emotional and cognitive factors together on preemptive behavior at multiple levels (behavioral, physiological and neurological). Finally, recent research has started using the cultural values (e.g., individualism and collectivism) at the national level to explain cross cultural differences in aggression (Archer, 2001; Bergeron and Schneider, 2005; Forbes et al., 2009). Individuals endorsing an individualistic orientation tend to emphasize individual rights and expect their members to assert and defend these rights (Triandis, 1995). In contrast, individuals endorsing a collectivistic orientation tend to value obligations to others, the avoidance of conflict, and the maintenance of social harmony (Triandis, 1995; Nisbett, 2003). Because of their strong emphasis on the avoidance of conflict and the maintenance of social harmony, it seems reasonable to expect that aggression and violence less common in collectivistic societies than in highly individualistic societies. In the future, it could be further explored whether cultural values can influence individuals’ preemptive behavior and whether individuals in collectivistic societies report higher level of hope, and then make less preemptive behavior than those in individualistic societies.

In summary, individuals are more likely to initiate preemptive behavior when they perceive a potential threat from the other in interpersonal interaction situations. Both fear and hope mediated the causal relationship between threat and preemptive behavior, but the roles were reversed, with fear increasing preemptive behavior and hope decreasing preemptive behavior. This study has important theoretical implications for a deeper understanding of the dynamics of interpersonal conflict, as well as helping people find solutions to the problem of defensive aggression, and thus has some application to improving people’s wellbeing.

## Data Availability Statement

The original contributions presented in the study are included in the article/supplementary material, further inquiries can be directed to the corresponding author.

## Ethics Statement

The studies involving human participants were reviewed and approved by the College of Teacher Education Ningbo University. The patients/participants provided their written informed consent to participate in this study.

## Author Contributions

LL, XS, and YL: development of ideas and writing of manuscript. All authors contributed to the article and approved the submitted version.

## Conflict of Interest

The authors declare that the research was conducted in the absence of any commercial or financial relationships that could be construed as a potential conflict of interest.

## Publisher’s Note

All claims expressed in this article are solely those of the authors and do not necessarily represent those of their affiliated organizations, or those of the publisher, the editors and the reviewers. Any product that may be evaluated in this article, or claim that may be made by its manufacturer, is not guaranteed or endorsed by the publisher.
